# Polyamines: Predictive Biomarker for HIV-Associated Neurocognitive Disorders

**DOI:** 10.4172/2155-6113.1000312

**Published:** 2014

**Authors:** Salim Merali, Carlos A. Barrero, Ned C. Sacktor, Norman J. Haughey, Prasun K. Datta, Dianne Langford, Kamel Khalili

**Affiliations:** 1 Department of Pharmaceutical Sciences, Temple University School of Pharmacy, Philadelphia, Pennsylvania, USA; 2 Department of Neurology, Johns Hopkins Memory and Alzheimer’s Disease Treatment Center, Johns Hopkins University School of Medicine, Baltimore, Maryland, USA; 3 Department of Neurology, Richard T Johnson Division of Neuroimmunology and Neurological Infections, Johns Hopkins University School of Medicine, Baltimore, Maryland, USA; 4 Department of Neuroscience, Center for Neurovirology, Temple University School of Medicine, Philadelphia, Pennsylvania, USA

**Keywords:** HIV-1, AIDS, Polyamines, Central nervous system, HIV associated neurocognitive disorders

## Abstract

**Objectives:**

Spermidine/spermine-N^1^-acetytransferase (SSAT) is the key enzyme in the catabolism of polyamines that are involved in regulating NMDA functioning. Over expression of SSAT leads to abnormal metabolic cycling and may disrupt NMDA receptor signaling. In fact, the HIV protein Tat induces neurotoxicity involving polyamine/NMDA receptor interactions. Thus, we investigated abnormal polyamine cycling in HIV+ participants with varying degrees of HIV-associated neurocognitive disorders.

**Methods:**

Acetyl-polyamine (SSAT products) levels were assessed by HPLC in CSF from 99 HIV-infected participants (no cognitive impairment (NCI, n=25), asymptomatic neurocognitive impairment (ANI, n=25), mild cognitive and motor disorders (MCMD, n=24), and HIV-associated dementia (HAD, n=25)). Polyamine levels in brain tissues from a subset of participants (uninfected (n=3), NCI (n=3), and MNCD (n=3)) were also assessed. Human primary astrocytes expressing HIV Tat were assessed for levels of the SSAT activity.

**Results:**

Activation of the polyamine catabolic enzyme, SSAT increases polyamine flux in brain and CSF of HIV infected individuals with HIV-associated neurocognitive disorders. CSF levels of acetylated polyamine increase with the degree of HAND severity as indicated by significantly increased acetylpolyamine levels in HAD participants compared to NCI and ANI (p<0.0001) and between MCMD and NCI and ANI (p<0.0001). *In vitro* studies suggest that the HIV protein Tat may be responsible in part for astrocyte-derived acetyl polyamine release.

**Interpretation:**

Our data suggest that polyamine metabolism may play a pivotal role in the neurodegeneration process among HAND patients. Changes in polyamine flux may serve as a potential predictive diagnostic biomarker for different severities of HAND.

## Introduction

Soon after primary infection, HIV is disseminated in the central nervous system (CNS) where productive replication in brain macrophages and microglia and limited expression of the viral genome in astrocytes may cause an array of toxic events that contribute to HIV-associated neurocognitive disorders (HAND) [1]. The presence of HIV and expression of viral proteins, even at low levels, in the brains of HIV patients taking combined antiretroviral therapy (cART) is often associated with neurocognitive disorders. It is estimated that over 50% of HIV-infected individuals with low viral load and high CD4 cell counts exhibit some form of HAND, and it is suggested that cART may be at least partially responsible for these impairments [2]. Based on the severity of disease, HAND is divided into three classes: asymptomatic neurocognitive impairment (ANI), mild cognitive and motor disorders (MCMD), and HIV associated dementia (HAD) [3,4]. Some HIV patients however, have no neurocognitive impairment (NCI). There are no molecular diagnostic biomarkers for different classes of HAND and diagnosis is mostly based on exclusion of other possible causes accompanied by neurological exam, neuropsychological tests, and brain MRI. Pathologically, HIV infection usually impacts cortical and subcortical regions and in the case of HIV encephalitis (HIVE) neuronal loss, astrogliosis, infiltrating macrophages, microglial nodules and multinucleated giant cells may be observed [5,6].

At subcellular levels, HIV-associated neuronal injury occurs indirectly by cytokines, chemokines and viral proteins such as Tat, gp120. Viral factors are neurotoxic to CNS cells through a variety of signaling pathways involving TNF-α, NMDA, AMPA and others [7,8]. Endogenous polyamines (putrescine, spermidine, and spermine) are known for modulating NMDA receptor function and early studies demonstrated that HIV Tat-induced neurotoxicity involves the interaction between polyamines and NMDA receptors [1,9]. Besides the effect on neurons, the polyamines, especially spermine, enhance astrocyte coupling through gap junctions [10]. Importantly, spermine accumulates almost exclusively in glial cells but not in neurons [11,12]. Therefore, these polyamines likely play a central role in astrocyte function.

Intracellular levels of polyamines are tightly regulated by homeostatic interactions between the anabolic and catabolic components of their metabolism. Spermidine/spermine-N^1^-acetytransferase (SSAT) is the key enzyme in the catabolism of polyamines and catalyzes the transfer of acetyl groups from acetyl-CoA onto the intracellular polyamines, spermidine or spermine. Acetylation reduces the positive charges on these molecules, alters their binding activity and renders them susceptible to cellular excretion and/or catabolism [13]. Importantly, acetylation also alters their ability to activate homeostatic responses. In mammalian cells, SSAT is tightly regulated and is highly inducible by polyamines [14]. The importance of SSAT in regulating polyamine homeostasis is indicated by its very short half-life, which is of the order of 20 min; this allows the cell to rapidly change enzyme and polyamine levels [15]. SSAT levels can also be induced by a variety of other stimuli including HIV Tat. These increases in SSAT are regulated by translational control mechanisms [16,17].

Recently, we reported the ability of SSAT to control polyamine flux through the metabolic pathway by showing that the over expression of SSAT leads to futile metabolic cycling [18]. However, the existence of this polyamine cycle and its consequences in patients with HAND have not been determined. Here we show that polyamine flux in astrocytes contributes to the elevation of the acetylated polyamines in the CSF of subjects with varying severities of HAND.

## Patients and Methods

### Human CSF and brain tissue samples

We obtained CSF samples (n=99) from the Johns Hopkins National Institute of Mental Health Center for Novel Therapeutics of HIV-associated Cognitive Disorders and CNS HIV Anti-Retroviral Therapy Effects Research (CHARTER) of the University of California at San Diego both under protocols approved by the respective Institutional Review Boards. The subjects’ demographic information is shown in Table 1. Participants included 75% male and 25% female, and the CD4+ T-cell counts of all participants ranged between 381–476 cells/mm [3]. All subjects were African-American with the exception of three female subjects in the no Neurocognitive Impairment (no-NCI) group and one female subject with HAD.. Among the participants, approximately 57% were HCV + and co-infection was evenly distributed among all HAND groups. Participants in this study were not current substance abusers. Additionally, we obtained frozen frontal cortex brain samples from 9 subjects (No-HIV, n=3; no-NCI, n=3; MCMD, n=3) from the Neuro AIDS Tissue Consortium (NNTC) in accordance with Temple University Human Subjects Protections and the Institutional Review Board.

### Primary astrocyte cultures

We obtained human primary astrocytes the Comprehensive NeuroAIDS Center at Temple University. Briefly, fetal brain tissue (gestational age, 16–18 weeks) was obtained from elective abortion procedures performed in full compliance with the National Institutes of Health and Temple University ethical guidelines. Tissue was mechanically and enzymatically dissociated, and astrocyte cultures were obtained by the orbital shaking method to remove other glial cells types. Astrocytes were cultured in growth medium containing 10% FBS until confluent. These astrocytes were transduced with adeno-null or Adeno-Tat [19]. In brief, complete media from primary astrocytes (3×10^6^ cells) grown in 10 cm tissue culture plate was removed and then treated with Adenovirus (Null or Tat) at an MOI of 5.0 plaque forming units per cell re-suspend in 1 ml of Optimem medium. After one hour of incubation at 37°C, complete cell culture medium was added and cells incubated for 24h. An additional control included un-transduced astrocytes. The medium from these cell cultures was saved and cell were washed in 1xPBS and harvested for polyamine, acetyl polyamines, SSAT and acetyl-CoA analyses. In addition the medium was analyzed for acetyl polyamines content.

### Analysis for polyamines and acetylated polyamines

The polyamines spermidine, spermine, and putrescine and acetylspermidine were analyzed by HPLC using a fluorescence tag previously shown to react with primary and secondary amines. This method utilizes an activated carbamate, N-hydroxysuccinidyl-6-aminoquinoyl carbamate (AccQ.Fluor), to derivatize the primary and secondary amines, providing stable and highly fluorescent adducts. We have previously demonstrated that analysis based on this pre-column derivatization method is quantitative, reproducible, linear and sensitive to 660 μmol [20]. Briefly, the biological samples were deproteinized by heating in a boiling water bath for 2 min. The samples were clarified by centrifugation at 2,000 × g for 5 min and the final supernatant was defined as the biological extract. Protein assays were conducted with the BioRad (Melville, NY, USA) dye binding assay using bovine serum albumin as the standard. The biological extracts (30 μl) were added to the borate buffer (0.2 M sodium borate, 1 mM EDTA, pH 8.8) for a final volume of 90 μl. The AccQ.Fluor reagent (10 μl) was added, and the samples were mixed and incubated in a 55°C water bath for 20 min to promote derivatization. For separation, the Waters HPLC system included a separation module (Model 2790) and a multi fluorescence detector (Model 2475). A 5 μm silica particle C8 Microsorb-MV column (150×4.6 mm I.D.) with a pore size of 100 Å was used. Fluorescence excitation was at 250 nm and emission was detected at 395 nm. The mobile-phase elution gradient was conducted as previously described [19]. All changes in the mobile phase were linear from one composition to the next. The flow-rate was 1.0 ml per min. All the analyses were performed at room temperature. The system was controlled and data were collected using Waters Millennium software. The peak-area values were calculated by the Millennium software.

### SSAT activity measurements

The SSAT activity in biological extracts was calculated by measuring the formation of the acetylspermidine from the added substrates, acetyl Coenzyme A and spermidine, as previously described [21,22]. The AccQ. flour tagged substrates and the products were separated by reverse phase HPLC and the amount of acetylspermidine was quantitated by integrating the area under the peak as described above for polyamine analysis. Briefly, the brain tissue in NKP buffer (2.68 mM KCl, 1.47 mM KH_2_PO_4_, 51.1 mM Na_2_HPO_4_, 7.43 mM NaH_2_PO_4_, 62 mM NaCl, 0.05 mM CaCl_2_, 0.05 mM MgCl_2_) was ultrasonicated for 5 min at 40 Watts and 70% duty cycle (Heat System, Ultrasonics Inc., Plainview, NY., USA). A cytosolic fraction was prepared by centrifugation of the homogenate at 100,000×g for 1 hr at 4°C. An aliquot of the supernatant was retained for Bradford protein assay. Spermidine (150 μM) was added to 40 μl supernantant and the enzyme reaction was initiated by addition of 250 μM of acetylCoA at 37°C. The samples were incubated for 10 min and the reaction was terminated by heating in a boiling water bath for 2 min. The sample was clarified by centrifugation at 5,000 × g for 5 min. The supernatant was assessed by HPLC analysis using AccQ. Fluor reagent as previously described [20].

### Acetyl-CoA quantitation using capillary electrophoresis

For acetyl-CoA measurements, primary astrocytes were lysed and processed using solid-phase extraction as previously described [23,24]. Extracts were analyzed on a Beckman P/ACE MDQ capillary electrophoresis system equipped with a photodiode array detector and an uncoated fused silica CE column. The column was 75 μm in diameter (inner) and 60 cm in length, and there was 50 cm from the inlet to the detection window. The column was preconditioned with 1 M NaOH and Milli-Q water for 10 min each at 20 p.s.i. The column was then equilibrated with 100 mM NaH_2_PO_4_ running buffer containing 0.1% α-cyclodextrin (pH 6.0) for 10 min. After each run, the capillary was rinsed with 1 M NaOH, Milli-Q water, and running buffer for 2 min each. The injection was conducted hydrodynamically at a pressure of 0.5 p.s.i. for 10 s. The injection volume was calculated using the CE Expert Lite software from Beckman. The separation voltage was 15 kV at a constant capillary temperature of 15°C. To establish standard calibration curves, solutions containing acetyl-CoA and the internal standard (isobutyryl-CoA, 41 nM) were prepared at concentrations ranging from 1 to 200 nM. Standards were processed as described above for cell lysates and suspended in 10 μl of water. Coenzyme was monitored with a photodiode array detector at the maximum absorbance wavelength (253.5 nm). Data were collected and processed using Beckman P/ACE 32 Karat software version 4.0. Cellular acetyl-CoA levels were expressed as nmoles/mg protein.

### Statistical analysis

All of the data were analyzed using Prism 6 (GraphPad Software Inc., La Jolla, CA, USA). The significant differences between the cohorts were assessed by Kruskal-Wallis and Dunn’s post multiple comparison tests. The Mann-Whitney U test (with Bonferroni correction) was used to compare the differences between the two groups. We elected to use nonparametric tests for the clinical data because of the skewed data distribution. For the parametric data, we used one-way ANOVA with post hoc Tukey-Kramer test to compare the groups. *in vitro* assays, we used the unpaired *t* test; the results were expressed as the mean ± SEM.

## Results

### SSAT driven polyamine flux is increased in brain samples from subjects with HAND

**Figure 1:**
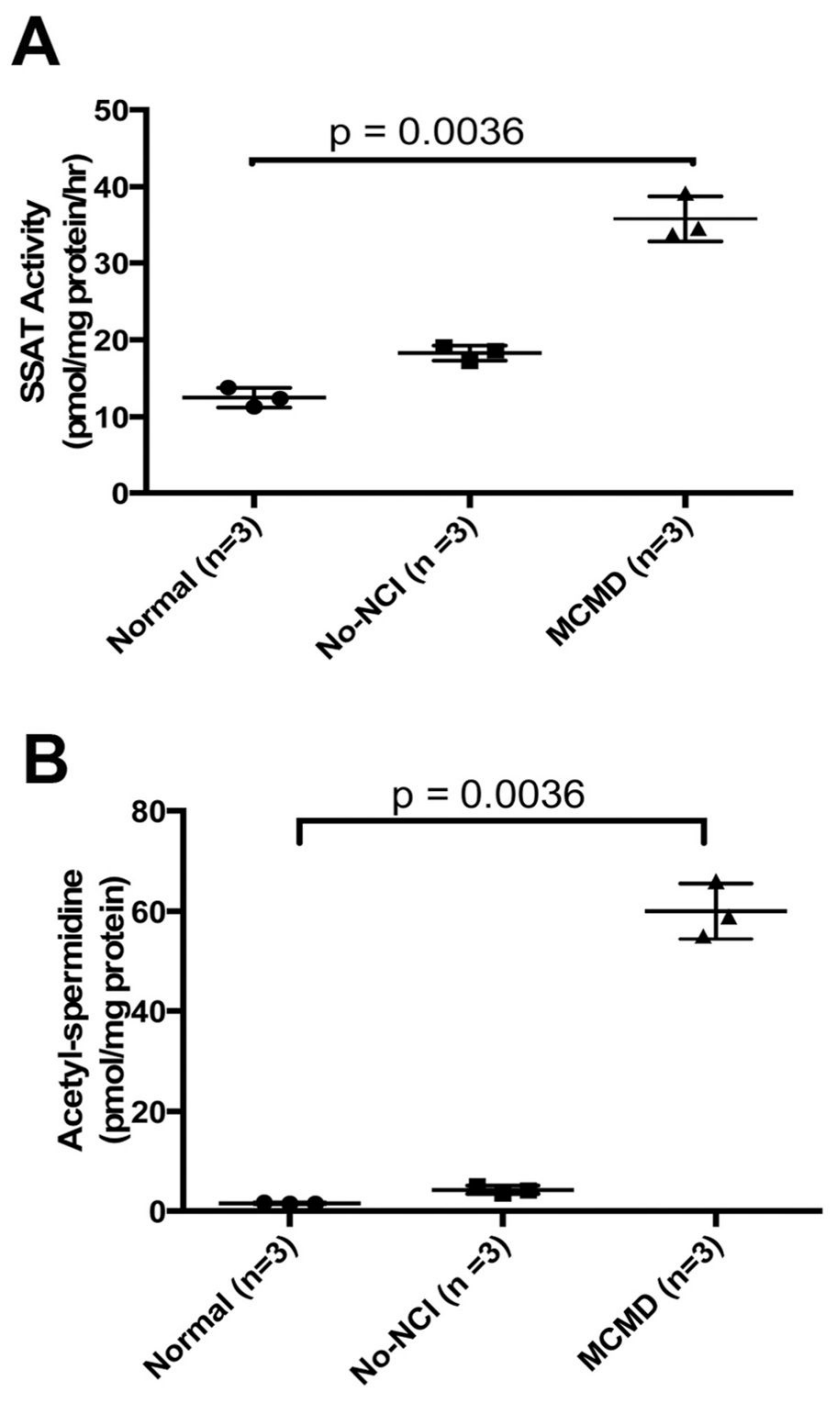
**A) SSAT activity is elevated in the lysates from the brains of patients with HAND.** A Kruskal-Wallis test was used to compare the groups. The mean differential between SSAT activity in the brains of MCMD as compared to No-HIV or HIV with NCI in pmol/mg protein/hr is (mean ± SD) 35.80 ± 2.972, 12.50 ± 1.25, 18.30 ± 0.985, respectively. **B) Acetyl-spermidine levels are elevated in the lysates from the brains of patients with HAND**. A Kruskal-Wallis test was used to compare the groups. The mean differential between acetyl-spermidine levels in the brain of MCMD as compared to No-HIV or HIV but NCI in pmol/mg protein is (mean ± SD) 60.00 ± 5.57, 4.27 ± 0.86, 1.56 ± 0.12, respectively.

Microarray studies show an increase in SSAT gene expression in response to HIV Tat over-expression in immature dendritic cells [25]. However, very little is known about enzymatic activity of SSAT in the brains of patients with HAND. To address this gap in knowledge, we measured SSAT activity in brain lysates from HIV patients with MCMD (n=3) and compared them to subjects with no-NCI (n=3) or normal no-HIV controls (n=3). Significant elevation of SSAT activity was detected in MCMD (Figure 1A). Since an increase in SSAT activity could trigger an increase polyamine metabolic flux [18], we tested this possibility and showed a significant increase in the levels of acetylspermidine in MCMD subsets in comparison to those from no-NCI and normal control (Figure 1B). Interestingly, the level of polyamine remained unchanged, indicating that polyamine flux is enhanced (Table 2A). Although, in this proof of principle study the group sizes were small, they do provide us with the results to support our hypothesis.

### Acetylpolyamines are released from astrocytes

**Figure 2:**
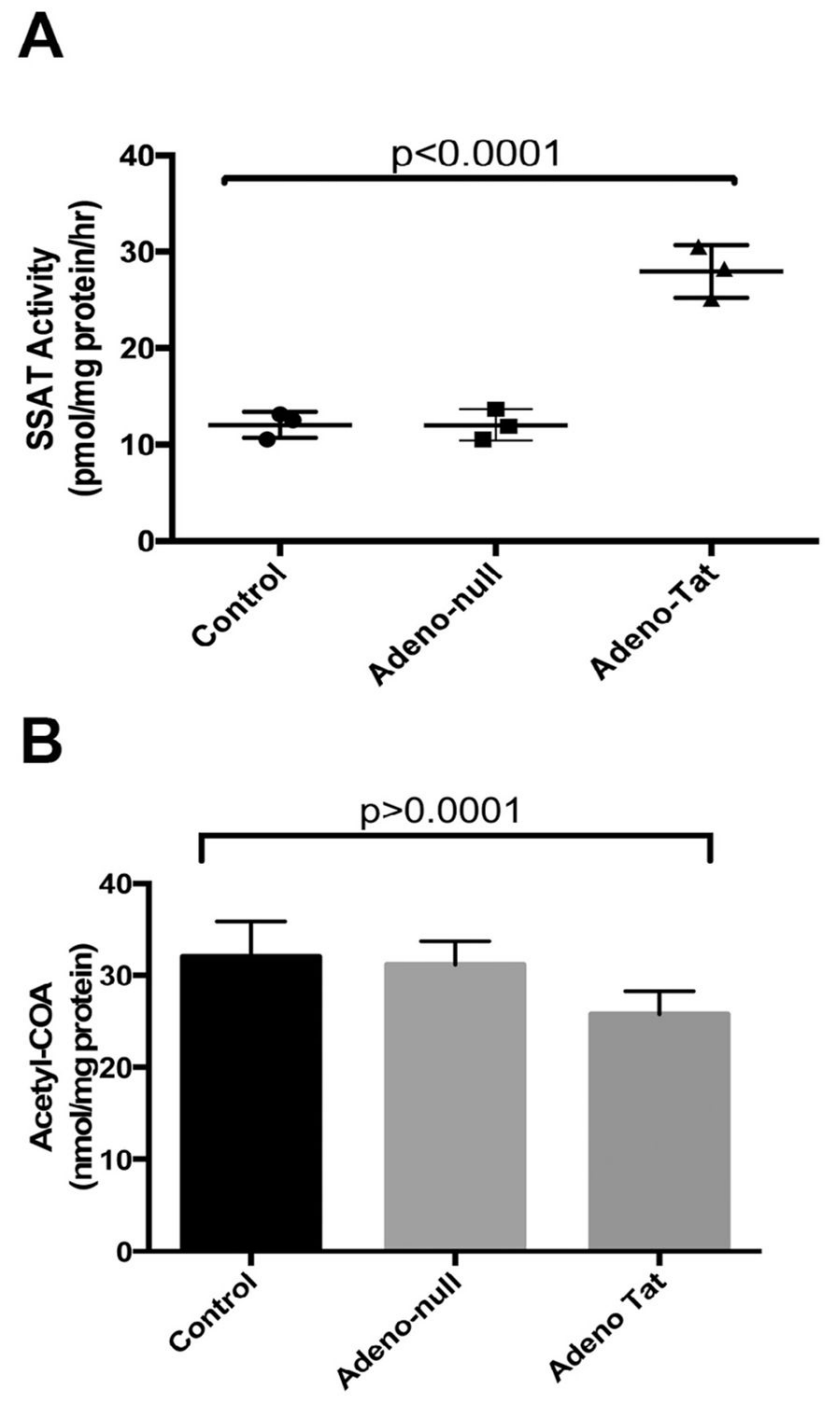
**A) SSAT activity in human primary astrocytes transduced to express HIV Tat.** A one-way ANOVA with Tukey-Kramer test was used to compare the groups (n =3). The mean differential between SSAT activity in the astrocytes transduced with HIV-Tat as compared to untransduced or transduced with Adeno-null in pmol/mg protein/hr are 27.93 ± 2.71, 12.03 ± 1.36, 12.03 ± 1.60, respectively. **B) Acetyl-CoA pools in primary astrocytes expressing HIV Tat**. A one-way ANOVA with Tukey-Kramer test was used to compare the groups. As consequence of polyamine flux, the acetyl-CoA levels are decreased in astrocytes expressing HIV Tat as compared to empty plasmid controls.

Astrocytes are known to play a significant role in the neuropathology of HAND. Hence, we investigated whether polyamine flux can trigger the release of acetylated polyamines from human primary astrocytes expressing HIV Tat. Our results show that the expression of Tat increased SSAT activity by approximately 3-fold in human primary astrocytes compared to untransduced astrocytes or astrocytes transduced with adeno-null (Figure 2A). Similar to brain tissue in HAD patients, the increase in SSAT activity may have contributed to increased polyamine metabolic flux, resulting in the decrease in the SSAT substrate acetyl-CoA and unchanged levels of polyamines. We tested these two possibilities by measuring acetyl-CoA levels and polyamines [24] in the same lysates used for measuring SSAT activity. The high-pressure capillary electrophoresis analysis of acetyl-CoA pools showed about 25% decrease in this SSAT substrate when the primary astrocytes were transduced to overexpress HIV Tat as compared to null-transduction and normal controls (Figure 2B). As expected, polyamine levels were not significantly changed in Tat expressing astrocytes compared to controls (Table 2B). The acetylation of polyamines decreases their positive charge, thereby increases the possibility for export of polyamines from cells. To evaluate this possibility, we quantitated the acetylated spermidine and acetylated spermine levels in the media of the cells transduced with HIV Tat and controls. Tat expression enhanced the level of acetylation of both spermidine and spermine compared to the controls (Table 2C). Taken together, these data support our hypothesis that Tat-induced increase in SSAT ratchets the polyamine flux and causes an increase in the acetylated polyamines and a decrease in the acetyl-CoA.

### Acetylated polyamine levels are increased in the CSF of the patients with HAND

**Figure 3:**
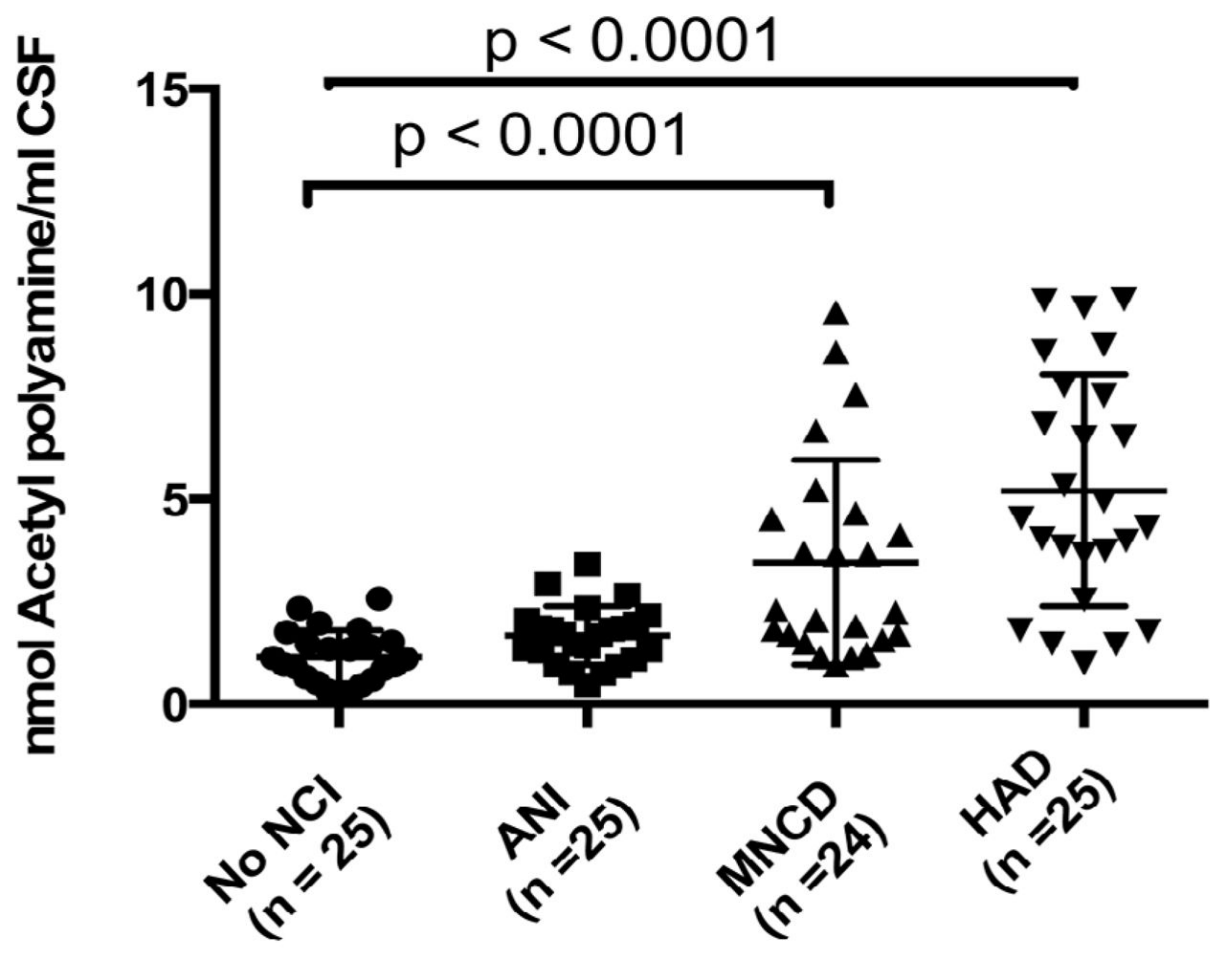
Acetylpolyamine levels in the CSF of the validation study (n =99) consisting of No-NCI, ANI, MCMD, HAD Acetylspermine levels are significantly elevated in MCMD and HAD groups compared to the no NCI and ANI groups (Kruskal-Wallis test).

Because our data showed an increase in the acetylated polyamine pools both in the brain tissue of patients with MCMD and in the media of HIV Tat transduced astrocytes, we hypothesized that the levels of acetylated polyamines would also be increased in the CSF of patients with HAND. To test this hypothesis, we assessed acetyl-polyamine levels in the CSF of three groups of subjects with different HAND severity i.e. ANI (n=25), MCMD (n=24) and HAD (n=25) and compared them to no NCI (n=25) HIV+ participants. All the groups totaling 99 participants were matched for age, gender; race and CD4+ T cell count (Table 1). The results show significant increases in acetylpolyamine levels in HAD compared to NCI and ANI (p<0.0001) and between MCMD and no NCI and ANI (p<0.0001) (Figure 3). Collectively, these results suggest that the levels of acetylated polyamine increase with the degree of HAND severity in CSF.

## Discussion

**Figure 4:**
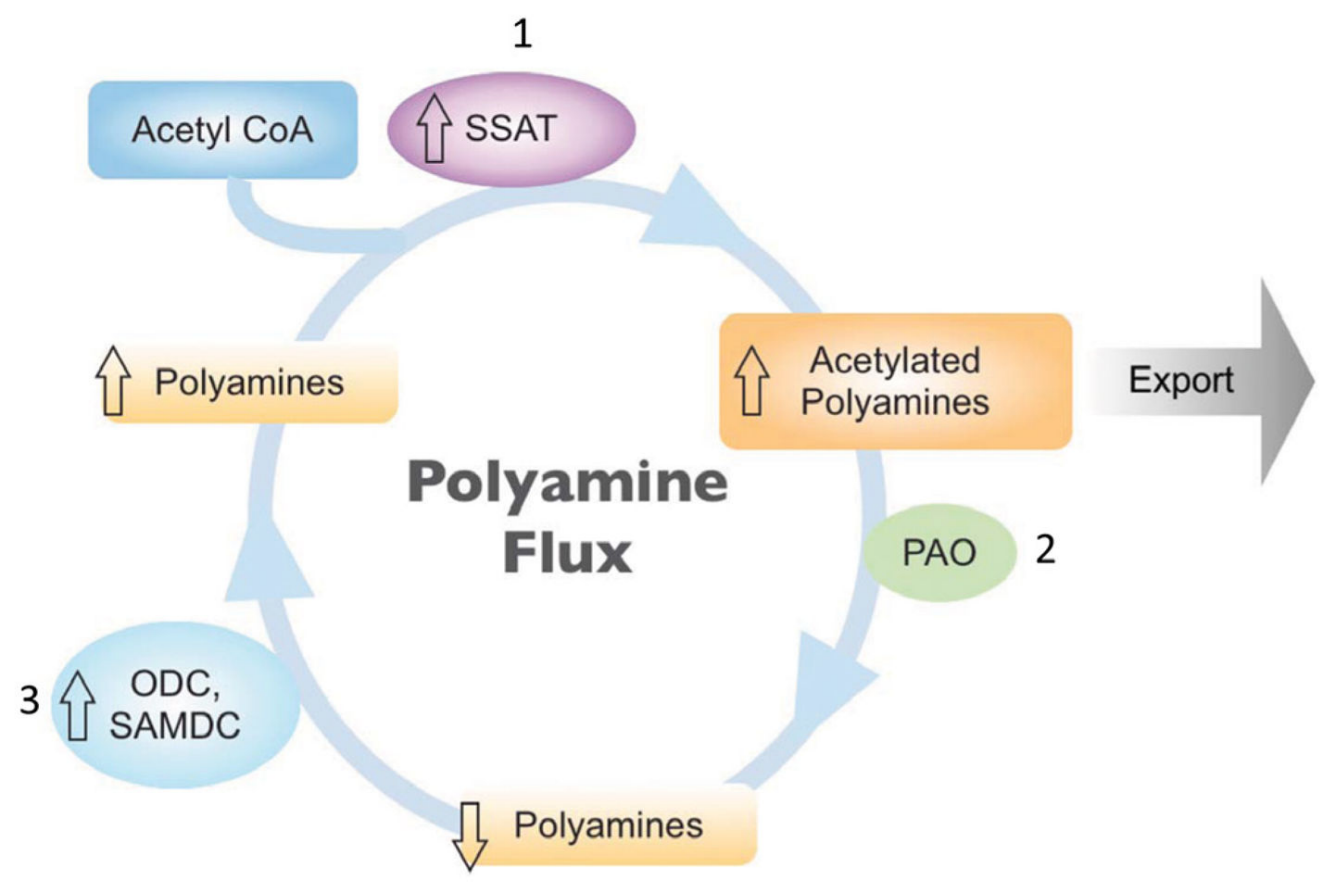
Proposed metabolic ratchet model for polyamine flux Increased SSAT initiates polyamine flux by acetylation of polyamines, which facilitates their export from the cell. Acetylation eliminates the ability of polyamines to repress biosynthetic enzymes (ODC and SAMDC), which leads to a compensatory increase in polyamine biosynthesis. The restored polyamine pools are then available for SSAT acetylation and continuation of the cycle. When flux increases, substrate utilization (such as acetyl-CoA) and product accumulation (such as acetylated polyamines) also increase but the levels of polyamines do not change. Our results show that induction of SSAT in astrocytes by HIV Tat leads to depletion of acetyl-CoA pools and accumulation of acetylated polyamines, a critical consequence of increased cycling. **Abbreviations:** ODC, Ornithine Decarboxylase; SAMDC, S-Adenosylmethionine Decarboxylase; PAO, Polyamine Oxidase.

Polyamines are known to enhance HIV reverse transcription *in vitro* [26]. Furthermore, SSAT, a rate-limiting enzyme in polyamine catabolism has been implicated in HIV pathogenesis. For example, it has been reported that SSAT expression is elevated in the Flp-In TREx 293 cell line overexpressing the HIV protein Vpr [27]. Moreover, studies using the yeast two hybrid system and immature dendritic cells show that both Vif and Tat, respectively can modulate the SSAT activity to impact polyamine levels [25,28]. However, the status of SSAT and its metabolic products in brain tissue and CSF were not known. Here, we report for the first time, that SSAT activity is elevated in brain tissue from HIV patients compared to uninfected controls, and this elevation is potentiated in patients with HAND. Further, we show that the elevation of SSAT positively correlates with the levels of acetylated polyamines (Figure 1). Previous studies have shown that the increase in SSAT activity influences polyamine homeostasis by modulating polyamine metabolic flux [18] (Figure 4). The consequences of polyamine flux are to maintain polyamine levels at the cost of increased consumption of precursors i.e. acetyl-CoA, which continues as long as SSAT levels are above baseline. The flux also generates more products such as acetylated polyamines. Based on these findings and because neurotoxic insults by HIV have been shown to disrupt glial NMDA receptor/polyamine interactions, we hypothesized that the acetylated polyamines are elevated in the CSF of HAND patients. To test this hypothesis, we first investigated the possibility of polyamine flux in human primary astrocytes transduced with HIV Tat. We chose to investigate astrocytes because these cells are believed to have a significant role in the neuropathology of HAND [29]. Astrocytes have been shown to have a complex bidirectional relationship with adjacent neurons and they play neurotrophic and pro-apoptotic roles [30]. The concept of the tripartite synapse has been suggested in which astrocytes not only perform housekeeping functions but also sense neurotransmitter release through the coincident release of gliotransmitters such as calcium, glutamate, nitric oxide, polyamines and biogenic amines [31]. In patients with HIV, dysregulation of these molecules may play key roles in HAND [8,32]. Our *in vitro* data show that while polyamines levels were unchanged, acetyl-CoA levels are depleted and the acetylated polyamines are increased intracellularly and in the media of the astrocytes transduced with HIV Tat. Collectively, these data support the increase in the polyamine flux in astrocyte transduced with HIV Tat. It has been reported that increased flux is associated with a reduction in acetyl-CoA, an increase in glucose catabolism, and an increase in oxidative phosphorylation [24,33]. Our data support this concept, demonstrating that polyamine levels are not altered but acetyl-CoA levels are decreased. Because acetyl-CoA is a sensor of the rate of glucose metabolism [33], the alteration of acetyl CoA levels is likely to impact glycolysis and lactate production. In other words, the polyamine flux may shift the glucose metabolism towards pyruvate and away from lactate resulting in decreased in lactate levels. This effect is likely to impact the astrocyte-neuron lactate shuttle and cause neuronal metabolic dysfunction.

The data supporting enhanced polyamine flux both in the brain tissue of subjects with HAD as compared to normal controls and astrocytes transduced to over-express HIV Tat prompted us to investigate the levels of these acetylated polyamines in the CSF of patients with and without HAND. Our data show that acetylated polyamines are increased in patients with HAD compared to HIV patients with normal cognitive functions and that these changes correlated with the severity of HAND.

Several limitations exist within the current study. Although proof of principal has been established for the impact of Tat on astrocytes, since these cells were assessed in isolation, the potential effects from other cells in the brain and on neurons was not addressed. Future expanded studies will investigate the effects of Tat exposure on neurotransmitter release in the context of the tripartite synapse. A second consideration of the study relates to other diseases that may influence NMDA neurotransmitter levels that may be affected by polyamine flux and acetylation state. For example, major depressive disorder, Alzheimer’s, insulin deficiency and others are believed to influence this pathway in the CNS. Co-morbidities including metabolic disorders may influence this pathway, as well. However, to the best of our knowledge, patients included in this study were not diagnosed with diseases known to impact polyamine flux and acetylation. Together, enhanced levels of acetylated polyamine in HIV patients with more severe neurocognitive impairment, suggests a potential role for these metabolites in the neurodegeneration process among HAND patients. Thus, polyamine may serve as a potential predictive diagnostic biomarker for different severities of HAND.

## Figures and Tables

**Table 1:** Demographic and clinical presentation of HIV subjects in the validation group (n = 99) check the CD4+T-cell count. (cells/mm^3^).

HAND diagnosis Age, years	No NCI (n=25)	ANI (n=25)	MCMD (n=24)	HAD (n=25)
Mean ± SD	46.6 (5.1)	46.0 (3.9)	48.4 (5.2)	46.9 (5.3)
Median (range)	47 (37 – 60)	46 (39 – 52)	49.5 (39 – 57)	46 (35 – 58)
Gender				
Male (%)	18 (72)	17 (68)	18 (75)	17 (68)
Female (%)	7 (28)	8 (32)	6 (25)	8 (32)
Race				
African American	22	25	24	24
Caucasian	3			1
CD4 T cell count				
Mean ± SD	537.9 (281.3)	441.5 (228.1)	500.8 (260.9)	447 (229.9)
Median (range)	459 (20 –1012)	381 (174 –1052)	476.5 (25 – 1056)	402 (19 – 973)

Abbreviations used: HAND=HIV-associated neurocognitive disorders; No NCI= no neurocognitive impairment; ANI=asymptomaticneurocognitive impairment; MCMD=minor cognitive-motor disorder; HAD=HIV-associated dementia

**Table 2A:** Polyamine levels in the brain: No-HIV, n=3; NCI, n=3; and MNCD, n=3.

	Putrescine (pmol/mg tissue)	Spermine (pmol/mg tissue)	Sperm idine (pmol/mg tissue)
No-HIV	18 ± 4.1	162 ± 11	549 ± 31
no-NCI	20 ± 3.2	171 ± 15	538 ± 46
MCMD	35 ± 3.8	182 ± 24	522 ± 22

**Table 2B:** Effect of HIV Tat on polyamine levels. Results are presented from three independent experiments (mean ± SD).

	Putrescine (mM)	Spermidine (mM)	Spermine (mM)
Control	0.41 ± 0.15	0.79 ± 0.25	1.10 ± 0.21
Adeno-null	0.45 ± 0.32	0.82 ± 0.45	1.24 ± 0.35
Adeno-Tat	0.61 ± 0.39	0.75 ± 0.36	0.95 ± 0.33

**Table 2C:** The levels of acetylated polyamines in the media of primary astrocytes transduced with HIV Tat.

	Acetyl-Spermine (pmol/mg)	Acetyl-spermidine (pmol/mg)
Control	1.5 (DL)	**1.5 (DL)**
Adeno-null	1.5 (DL)	**1.5 (DL)**
Adeno-Tat	3.2 ± 1.6	5.2 ± 2.1

DL: Detection limit
